# Multi-Focus Image Fusion Based on Convolution Neural Network for Parkinson’s Disease Image Classification

**DOI:** 10.3390/diagnostics11122379

**Published:** 2021-12-17

**Authors:** Yin Dai, Yumeng Song, Weibin Liu, Wenhe Bai, Yifan Gao, Xinyang Dong, Wenbo Lv

**Affiliations:** 1 College of Medicine and Biological Information Engineering, Northeastern University, Shenyang 110169, China; sym18343540280@163.com (Y.S.); 2071225@stu.neu.edu.cn (W.L.); 2071205@stu.neu.edu.cn (W.B.); yifangao@stumail.neu.edu.cn (Y.G.); w1061142559@163.com (W.L.); 2 Engineering Center on Medical Imaging and Intelligent Analysis, Ministry Education, Northeastern University, Shenyang 110169, China; 3 School of Biomedical Engineering (Suzhou), Division of Life Science and Medicine, University of Science and Technology of China, Hefei 230026, China; 4 Wentworth College, University of York, York YO10 5NG, UK; xyd_707@163.com

**Keywords:** Parkinson’s disease (PD), deep learning, multi-focus image fusion

## Abstract

Parkinson’s disease (PD) is a common neurodegenerative disease that has a significant impact on people’s lives. Early diagnosis is imperative since proper treatment stops the disease’s progression. With the rapid development of CAD techniques, there have been numerous applications of computer-aided diagnostic (CAD) techniques in the diagnosis of PD. In recent years, image fusion has been applied in various fields and is valuable in medical diagnosis. This paper mainly adopts a multi-focus image fusion method primarily based on deep convolutional neural networks to fuse magnetic resonance images (MRI) and positron emission tomography (PET) neural photographs into multi-modal images. Additionally, the study selected Alexnet, Densenet, ResNeSt, and Efficientnet neural networks to classify the single-modal MRI dataset and the multi-modal dataset. The test accuracy rates of the single-modal MRI dataset are 83.31%, 87.76%, 86.37%, and 86.44% on the Alexnet, Densenet, ResNeSt, and Efficientnet, respectively. Moreover, the test accuracy rates of the multi-modal fusion dataset on the Alexnet, Densenet, ResNeSt, and Efficientnet are 90.52%, 97.19%, 94.15%, and 93.39%. As per all four networks discussed above, it can be concluded that the test results for the multi-modal dataset are better than those for the single-modal MRI dataset. The experimental results showed that the multi-focus image fusion method according to deep learning can enhance the accuracy of PD image classification.

## 1. Introduction

### 1.1. Background of Parkinson’s Disease

Parkinson’s disease (PD) is a common neurodegenerative disease in the middle aged and elderly. The symptoms differ for each person. The main characteristics of PD are resting tremors, muscle tonus or rigidity of the extremities, delayed movements, and postural balance disorders [1]. PD is primarily attributable to reduced levels of the neurotransmitter dopamine in the nigrostriatal system of the brain. Dopamine levels fall as the number of dopamine-producing cells in the brain decreases. At present, since most PD patients have no obvious clinical symptoms, it is difficult to make an accurate diagnosis only as per its clinical manifestations and a series of routine examinations [1]. In addition, the symptoms of the disease are similar to those of other diseases, causing misdiagnosis in the early stages to occur frequently. The number of nigrostriatal dopamine neurons is significantly reduced by the time most people are diagnosed with PD. At this point, patients miss out on optimal treatment at an early stage as their diseases have become severe [2]. As a result of this, timely detection of PD contributes to the rapid treatment and significant relief of symptoms [3].

The application of neuroimaging in the diagnosis of PD has become increasingly widespread in recent years. However, it has been found that the patient was diagnosed with PD when most of the neurons had degenerated [4]. Therefore, it is harder to recognize and diagnose PD only based on the clinical symptoms. The diagnostic accuracy of PD has improved owing to the many computer-aided diagnostic (CAD) techniques that have emerged in recent years.

There are currently many CAD methods for PD. There are several imaging methods when it comes to PD. First, Byeong [5] uses image processing technology and automatic segmentation methods to study the cortex. The global cortical of Parkinson’s patients atrophies compared with healthy people. The advantage of using this method is that it has high accuracy detection in the early period of PD. There are also machine learning methods available. Gabriel [6] uses voxel-based morphology (VBM) to extract the featured area of the magnetic resonance image (MRI). This is then used in the machine learning method to classify the area to achieve a high accuracy result. Deep learning techniques are available. Sivaranjini [4] used deep learning to classify MRI of PD. This also uses the transfer learning tool to train and test images to achieve high accuracy in test results. Because there are various diagnostic methods, there is no clear method in the diagnostic criteria of PD [7].

There are many diagnostic methods for PD at present. The result of CAD is much superior to other methods [8]. CAD has been developed in medical image diagnosis for an extended period. In early research, it has been found that CAD is more accurate than pathologists in diagnosing diseases [8]. Toltosa [9] shows that the clinical diagnosis has high uncertainty and minuscule values for detecting PD. However, CAD can improve the accuracy of the diagnosis significantly by using techniques such as imaging and genetic testing methods. Rizzo [10] shows that the clinical diagnosis of PD is not an idea since it requires neuroimaging to assist it. This is not optimal through a relevant literature review in the past 25 years. Pyatigorskaya [11] mentioned that CAD has made significant progress in the diagnosis of PD in the last 10 years. Heim [12] shows that MRI improves the accuracy of PD by summarizing the results of different MRI studies on PD. Heim [12] also summarized the application of single-modal images and multi-modal images in the diagnosis of PD. The results are significantly better when using a combination of different technologies than when using a single technology. Rojas’s [13] experiment has demonstrated that image fusion can improve the performance of images by using the fusion of brain imaging techniques on patients with PD or other diseases. Soltaninejad’s [14] experimental comparison concluded that the fusion of multi-modal data classification had higher accuracy than single-modal data classification. Dai [8] conducted comparative experiments and drew the conclusion that multi-modal images have better diagnostic effects than single-modal images. With all these test results, it can be determined that CAD has participated a significant role in diagnosing PD.

### 1.2. Current Situation of Convolution Neural Network Diagnosis

With the rapid growth of modern technology, deep learning has evolved with the speed of technology, and new convolutional neural networks appear frequently. Convolutional neural networks have a wide range of applications in various fields. The networks have made great achievements in the field of medical image analysis. In previous case studies on PD, the AlexNet network and MRI were used to diagnose PD with an accuracy of 88.9% [4].

This article uses AlexNet first to classify Parkinson’s image data sets for comparison. It then uses Densenet, ResNeSt, and Efficientnet networks to classify the same Parkinson’s image data sets. This allows the accuracy of these three networks for Parkinson’s image classification to be verified.

### 1.3. Image Fusion

Image fusion can be performed at different levels. Pixel-level fusion is the fundamental and most common fusion method [15]. Pixel-level image fusion is frequently used in remote sensing, medical imaging, and computer vision [16]. Medical image fusion is to match and fuse multiple images of the same area with different imaging modes to obtain more information [17]. In neuroimaging, MRI displays structural information while positron emission tomography (PET) shows lesion information. The technique of fusion between MRI and PET allows additional information to be extracted.

The fused image is more suitable for human vision or machine perception [15]. Image fusion can obtain more accurate information for clinical diagnosis, so it has good use value in medical diagnosis [18]. Yang [19] makes the operation more accurate with better results and increases the safety of the operation through the application of MRI and Computed Tomography (CT) fusion images for preoperative analysis and evaluation. Bi [20] used PET and CT fusion to make the segmentation results more accurate.

In early development on image fusion, spatial domain or transform domain methods were used commonly [21,22,23,24]. The spatial domain method is to process pixels directly. These methods include Averaging, Brovey Method, Principal Component Analysis (PCA), Intensity-Hue-Saturation (IHS) [22,23]. The transform domain method requires Fourier transform. The methods include Discrete Wavelet Transform (DWT), Stationary Wavelet Transform (SWT), Contourlet Transform (CT), Discrete Ripplet Transform (DRT) [22,23].

Omar [21] has systematically reviewed, compared, and analyzed the image fusion methods. Pyramid-based methods are commonly used in the field of image fusion. However, this method lacks flexibility and anisotropy. The DWT method can overcome the limitations of the pyramid-based method. Unfortunately, it has an offset variance, so the discontinuity of the source signal will cause bad results.

Bhataria [22] has reviewed both the fusion method of spatial domain and the transform domain. The PCA in spatial domain fusion provides a high information quality. However, it is extremely dependent on the dataset and it can lead to spectral degradation. Furthermore, the spatial domain fusion method is complicated and not time efficient. The DWT in the transform domain fusion method has the characteristics of critical sampling, localization, and multi-resolution. However, DWT cannot accurately display the edges of an image and it cannot provide directionality. DRT has several characteristics, such as multi-scale, directionality, and localization. However, DRT does not provide multi-resolution.

Vora [23] reviewed and compared PCA, DWT, and SWT. The following conclusions are obtained: PCA has ambiguity, but this can be resolved by transforming domain technology. Although the peak signal-to-noise ratio (PSNR) of SWT is lower and the mean square error (MSE) is higher, it is better than DWT.

MNB [25] stated that the PCA does not have a fixed set of basis vectors and relies on the initial dataset. This point is consistent with the view of [22]. The two methods of DWT and SWT are similar. However, the downsampling process of SWT is suppressed, making the SWT translation-invariant, making DWT a better method.

Dulhare [24] reviewed and compared the image fusion methods in the spatial domain, transform domain and deep learning. The review includes several experiments, which the result has proved that the image fusion methods that’s based on deep learning are superior to the methods of spatial and transform domain.

In modern history, image fusion technology based on deep learning has attracted the attention of researchers. The deep learning model can automatically extract the most effective pixel features. It also allows us to overcome the difficulty of manually designing a complex activity-level measurement and its fusion rules [26]. This method shows that image fusion technology has made a breakthrough.

Liu [27] used a multi-focus image fusion technique that is based on deep convolutional neural networks to fuse MRI T1 and T2 weighted images. This has returned outstanding results. Therefore, to increase the quality and amount of information, this research paper used the same deep learning method to fuse MRI and PET images [17]. PET-MRI fusion can improve pathology prediction by improving the accuracy of the region of interest (ROI) localization [28].

## 2. Methods

**Figure 1:**
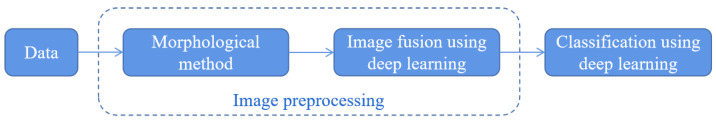
Experimental process.

This experiment collects the data that needs to be pre-processed first. These methods of pre-processing include morphological image processing and multi-focus image fusion based on deep learning. It is then MRI and PET images fused. Finally, the experiment uses convolutional neural networks to classify the images, as shown in Figure 1.

### 2.1. Data

The data used in this article is from the Parkinson’s Progression Markers Initiative (PPMI) database. Since the middle and late stages of PD have undergone substantial lesions, these treatments will be ineffective against patients who have middle or late stages of PD [29]. Therefore, the data selected in this article only consists of the early stages of PD. Early diagnosis is helpful to control the development of the disease.

**Figure 2:**
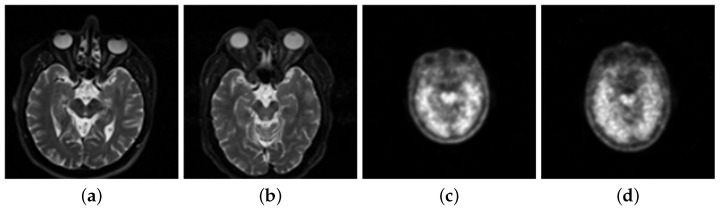
(**a**) Magnetic resonance image (MRI) of patients. (**b**) MRI of normal people. (**c**) Positron emission tomography (PET) image of patients. (**d**) PET image of normal people.

The data set for the experiment consists of 206 patients and 230 healthy people’s MRI with a weight of T2 from PPMI. This is demonstrated in Figure 2a,b. The paper then uses 621 patients’ images and 751 healthy people’s images as the single-modal MRI data. This data set is from the experiments’ total MRI. The paper selected the PET neuroimages with marker 18-FDG, as shown in Figure 2c,d.

### 2.2. Data Preprocessing

#### 2.2.1. Morphological Methods

**Figure 3:**
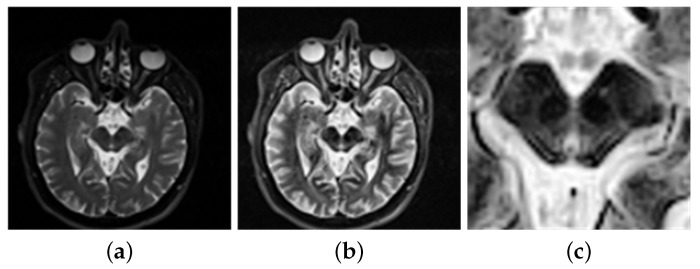
(**a**) Before image enhancement. (**b**) After image enhancement. (**c**) Region of interest.

The data requires processing before entering the network classification. Hasford [30] uses imadjust to process these images to enhance the quality of the fusion images. These images are then fused by the proposed fusion method. The accuracy of the fusion algorithm is then evaluated, and a satisfactory result is returned. In addition, Veronica [31] used imajust for contrast enhancement of the images. Then the neural networks are used to classify the feature set of the lungs. This will return an accurate result. Kadam [32] used imadjust to enhance the images on a brain tumor. It has displayed a good result, hence allowing the method to be used for tumor detection. In order to get a clearer image, this paper uses the imadjust function to enhance the brightness of the image. The brightness value of the original image is mapped to the new image, achieving the contrast enhancement effect, as in Figure 3b.

Pyatigorskaya [11] demonstrates that the volume of the substantia nigra (SN) varies, which allows PD detection through investigations and research on the diagnosis of PD. Soltaninejad [14] selected the SN as the ROI to diagnose PD. This has obtained good results. Al-Radaideh [28] mentioned that PD is caused by the loss of neurons in the SN. These factors allow SN to be chosen as the ROI. Therefore, this experiment adjusts the image to the appropriate size and intercepts the ROI of 224×224 from the center, as shown in Figure 3c. The extracted regional features are more obvious, allowing them to be conducive to the training network and receive more accurate results.

PET imaging predominately displays lesion information. This method is highly sensitive to biomarkers in vivo at the molecular level, making it not able to provide accurate anatomical information [28]. This allows PET images to be commonly used in the differentiation, monitoring, and treatment of benign and malignant tumors. MRI provides a wide range of image contrast, high spatial resolution, and more comprehensive information about highly deformed soft tissues. Therefore, the training and testing of single-modal data are mainly used in single-mode MRI datasets [21,28,33].

#### 2.2.2. Image Fusion

At present, most image fusion algorithms operate at the pixel level [15]. The pixel-level fusion includes the steps of image preprocessing, registration, and fusion. The size of the ROI of the morphologically processed MRI and PET images is adjusted to be the same. Then, they are registered using the full automatic multi-modal image registration algorithm [34]. Next, the 224×224 ROI size is then extracted from MRI and PET images and then fused by using a multi-focus image fusion method based on a deep convolution neural network. Finally, the dataset containing 736 normal human images and 614 patient images has been generated.

The paper used a multi-focus image fusion method based on a deep learning convolutional neural network. The fusion method is a method of using a neural network to classify the focus. Liu [27] has been proved that medical image fusion can apply to this method.

**Figure 4:**
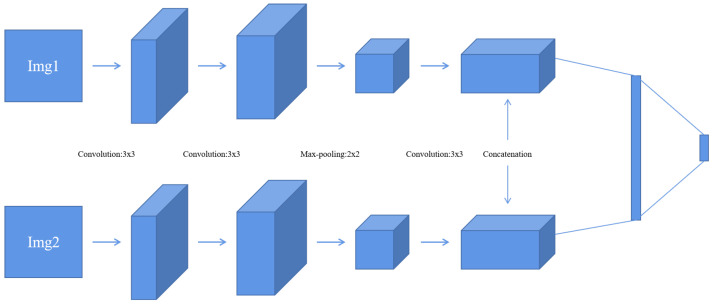
Convolutional neural network (CNN) model [27].

**Figure 5:**
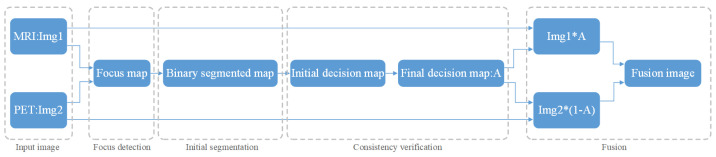
Process of image fusion.

This method uses the network model proposed in [27], as shown in Figure 4. The stochastic gradient descent (SGD) method is used to minimize the loss function. The batch size is set to 128. The weights are updated with the following rule [27]:

(1) $$\begin{array}{cc} \; & v_{i+1}\;=\;0.9\cdot v_{i}-0.0005\cdot \alpha \cdot w_{i}-\alpha \cdot \frac{\partial L}{\partial w_{i}}\\ \; & w_{i+1}\;=\;w_{i}+v_{i+1} \end{array}$$

where *v* is the momentum variable, *i* is the iteration index, α is the learning rate, *L* is the loss function, and $\frac{\partial L}{\partial w_{i}}$ is the derivative of the loss with respect to the weights at $w_{i}$. This method has the following steps, as shown in Figure 5:

1. Firstly, this experiment has pre-registered two images and they will be noted as img1 and img2. The images will be inputted into the neural network. This method compares the same position of the two images in the same group. It is then assigned a value between 0 and 1 to each coefficient point to represent the focusing characteristics of the image. The closer the points’ coefficient is to 1, the more focused the point is. This experiment then obtains a focused image [27].
2. This experiment uses a threshold of 0.5 to segment the focus image initially. Then, inside the focus map, the experiment marks the coefficient values greater than 0.5 as 1. Similarly, it also marks values less than or equal to 0.5 as 0. This allows the experiment to obtain a binary piecewise map A.
3. The next step is used for consistency verification. Deleting incorrect points in the binary segmented graph and extracting the maximum connected component of the binary map allows the initial decision diagram to be returned [27]. After extracting the diagram, the experiment uses Formula (2) to calculate the gray image of the original image, the initial decision graph, and the filtered operation results. Following this, the experiment obtains a final decision diagram [27].
4. Finally, the experiments fused the multiplication of MRI and the final decision map and the multiplication of PET and the final decision map complement into an image. Then, the experiment obtains a final fusion image that follows Formula (2) [15].
  (2) C(x,y)=A(x,y)Img1(x,y)+(1−A(x,y))Img2(x,y)

The multi-modal fusion data set was obtained by fusing MRI and PET images using a multi-focus image fusion method based on deep convolution neural networks. This includes focus detection, initial segmentation, consistency verification, and fusion processes [27].

### 2.3. Classification of Convolutional Neural Network

The current research on PD does not have documented Densenet, ResNeSt, and Efficientnent to classify PD images. This experiment intends to use the controlled variable method to use the Alexnet network as a comparative study. By using Alexnet to classify the PD image dataset, it can retrieve the results. The result is then compared with the results of Densenent, ResNeSt, Efficientnet to classify the same datasets. This paper compares all the results retrieved via different methods.

#### 2.3.1. Local Direct Connected Structure Densenet

**Figure 6:**
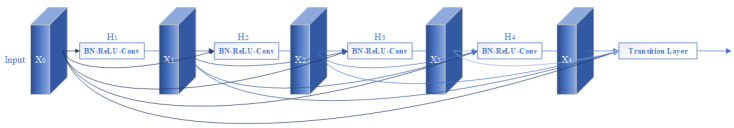
Network structure of Densenet [35].

ResNet uses a basic block and bottleneck structure. Its counterpart, Densenet, uses a connection structure. A dense block is shown in Figure 6. The input of each layer of Desenent is dependent on the output of all previous layers. This structure reduces the network parameters and makes the training significantly easier. Huang [35] showed that the Desenent network uses fewer network parameters in comparison to ResNet when both are trained with the same accuracy. Densenet introduces a direct connection from any layer to all subsequent layers. Because of this effect, this layer receives inputs from all previous layers as $X_{0},...,X_{l-1}$. Then, the outputs are the following:

(3) $$X_{l}=H_{l}\left(\left[X_{0},X_{1},....,X_{l-1}\right]\right)$$

where $\left[X_{0},X_{1},....,X_{l-1}\right]$ refers to the transition at layers 0,...,l−1 [35].

#### 2.3.2. Modular Network Structure Efficientnet

**Figure 7:**
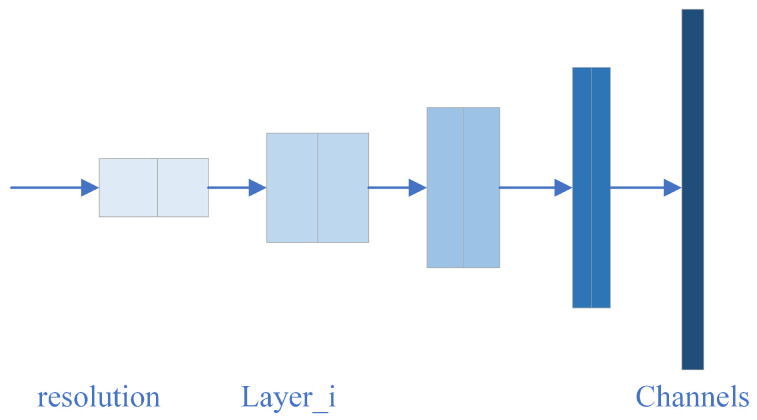
Network structure of Efficientnet [37].

Efficientnet has eight models, but the backbone of each model is the same. They mainly contain seven blocks, with each block containing several sub-blocks. The basic network structure is shown in Figure 7. The accuracy of training results will be different depending on the width, depth, and input resolution of the network. To improve this accuracy, expanding the width and depth of the network can be implemented, along with increasing the input resolution [36]. Since Efficientnet uses few parameters as inputs, it is highly efficient and will return satisfactory training results.

#### 2.3.3. Structures of Multi-Channels ResNeSt

**Figure 8:**
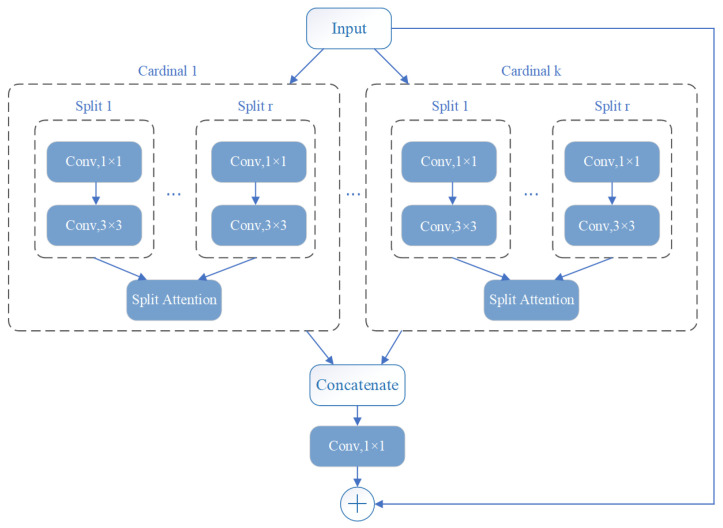
Network structure of ResNeSt [38].

ResNeSt is a modified network based on ResNet. It contains a split attachment block, which is a computational unit. ResNeSt divides the features into k groups, each labeled cardinal 1−k. Then each cardinal is divided into *r* groups. Thus, there are a total of G=k×r feature groups, as shown in Figure 8 [38].

Zhang [38] uses the proposed ResNeSt to compare with other neural networks in image classification methods. He concluded that ResNeSt obtains the highest accuracy rate. Better results are also obtained in object detection, instance segmentation, and sematic segmentation with comparison to other networks. This multi-channel format was used to improve efficiency and accuracy when compared to other networks. The ResNeSt network used in this paper is the ResNeSt50 model.

### 2.4. Training and Testing of Neural Networks

By using the Alexnet network with the three networks described above, testing and training can be conducted against single-modal MRI datasets and multi-modal datasets. The aim of the training and testing is to verify which modality of data is more suitable for the classification of PD images. This can lead to a conclusion on which network is more suitable for classifying PD images.

This paper uses 5-fold cross-validation to train and test the different image sets. Five-fold cross-validation means dividing the data into five distinct groups. This experiment uses one group as the input test set, while the other four groups of data are used for training. Then the experiment is repeated five times to make each copy of the data set into a test set. The final test accuracy is calculated by taking the average of five test accuracy. This approach prevents overfitting, therefore improving the stability of the model. Cross-validation is widely used in machine learning [39].

The three networks use the cross-entropy loss function to train the network and use adaptive momentum (Adam) to minimize the loss function. The learning rate for this network is 0.0001. The batch size is 10. The classification results are evaluated using metrics of Accuracy, Recall, Precision, Specificity, and $F_{1}$-Score. The specific formula is the following:

(4) $$\begin{array}{cccc} \; & Accuracy\; & = & \;\frac{TP+TN}{TP+FP+TN+FN}\\ \; & Recall\; & = & \;\frac{TP}{TP+FN}\\ \; & Precision\; & = & \;\frac{TP}{TP+FP}\\ \; & Specificity\; & = & \;\frac{TN}{TN+FN}\\ \; & F_{1}-Score\; & = & \;{\left(\frac{recall^{-1}+precision^{-1}}{2}\right)}^{-1} \end{array}$$

The receiver operating characteristic curve (ROC) and the confusion matrix were outputted to show the experimental results. An area under the curve (AUC) is also calculated to further demonstrate the results. The vertical axis of ROC is Recall, and the horizontal axis is:

(5) $$FPR\;=\;\frac{FP}{FP+TN}$$

Among them, TP is the number of true positive cases. If these cases are positive, the data displayed is also positive. TN is the number of true negative cases. If these cases are negative, the data displayed is also negative. FP is the number of false-positive cases, which shows that if these cases were negative, they would return positive results. FN (false negative) is the opposite of this, where several negative results have returned positive. This can be seen in Table 1. As the results are displayed, the confusion matrix can be output. For the binary confusion matrix, refer to Table 2.

The Alexnet, Densenet, ResNeSt, and Efficientnet networks were used to train and test the single modal MRI dataset. The MRI dataset contains 751 healthy human images and 621 PD images through the cross-validation method. The results are then recorded. The next step is to repeat the process but using multi-modal data. These datasets include 746 healthy human images and 614 Parkinson’s images.

## 3. Results and Discussion

### 3.1. Data Preprocessing

**Figure 9:**
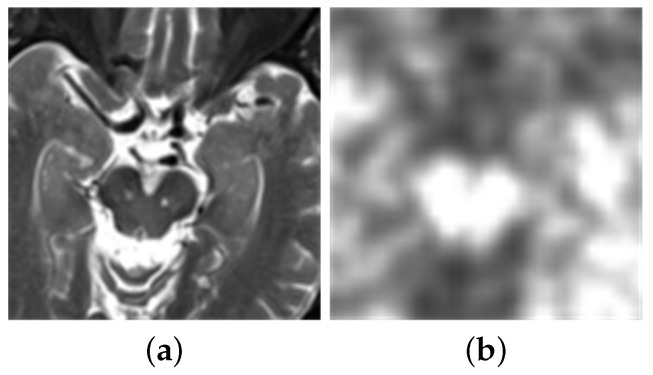
(**a**) Original MRI image. (**b**) Original PET image.

Following the method mentioned above, the multi-modal images used in the multi-focus image fusion method are based on a deep convolution neural network to fuse MRI and PET images. The MRI used is shown in Figure 9a, while PET images are shown in Figure 9b.

**Figure 10:**
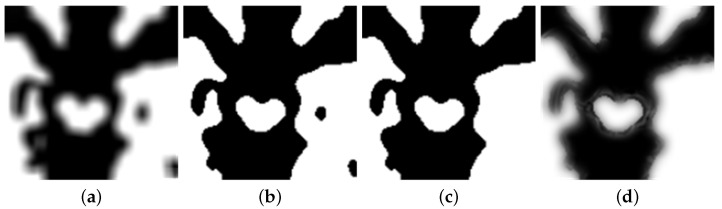
(**a**) Focus detection. (**b**) Initial segmentation. (**c**) Initial decision map. (**d**) Final decision map.

In this experiment, the two images are inputted into the network. Through the focus detection, the experiment has acquired the focus imagine, shown in Figure 10a. The binary image is obtained by initial segmentation of the focus image, as shown in Figure 10b. After consistency verification, the experiment obtains the initial decision map, followed by obtaining the final decision map, as shown in Figure 10c,d.

**Figure 11:**
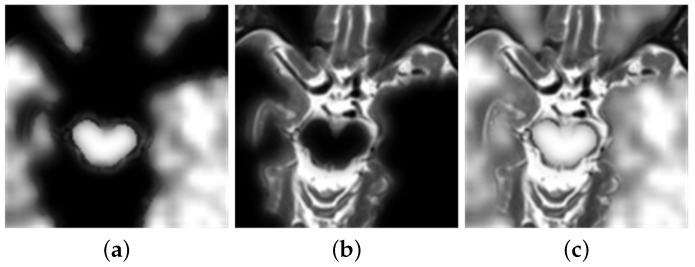
Image fusion. (**a**) The product of MRI and final decision map. (**b**) The product of PET and complementary set of final decision map. (**c**) Final fusion image.

Figure 11a depicts the multiplication of MRI and the final decision graph obtained from Formula (2). The multiplication of PET with the complement of the final decision graph is shown in Figure 11b. The final fusion image is shown in Figure 11c.

**Figure 12:**
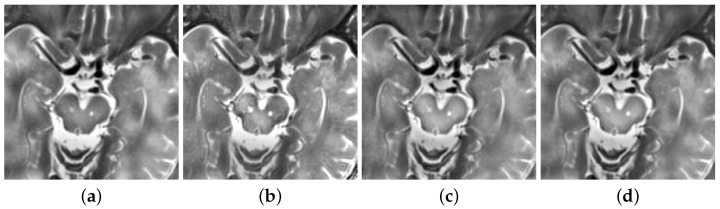
(**a**) Fusion results of Laplacian pyramid (LP). (**b**) Fusion results of ratio of low-pass pyramid (RP). (**c**) Fusion results of curvelet transform (CVT). (**d**) Fusion results of nonsubsampled contourlet transform (NSCT).

In this paper, Laplacian pyramid (LP), the ratio of a low-pass pyramid (RP), curvelet transform (CVT), and nonsubsampled contourlet transform (NSCT) methods are used for comparative research [40]. This article uses these methods to fuse MRI and PET, as shown in Figure 12.

By comparing the results of these fusion methods, the visual appearance effects of the LP, RP, CVT, and NSCT methods are not obvious. The relevant information of PET is also not obvious. However, the results obtained by using the deep learning method, shown in Figure 11c, can clearly distinguish the contours of MRI and PET. In addition, this experiment also uses several objective criteria to evaluate these fusion results. These fusion results include structural similarity index measure (SSIM), spatial frequency (SF), mutual information (MI), standard deviation (STD), and correlation coefficient (CC). Table 3 also shows these results.

The value of SSIM is between 0 and 1. The larger the value, the higher the degree of image fusion [41]. SF reflects the change of the image at a grey level. A larger value results in the image being clearer and the quality of the fusion image is better [42]. Similarly, the larger the MI, the more information can be obtained from the original image, causing the quality of the fusion to increase [43]. STD also reinforces the quality of fusion by increasing the amount of information contained in the image [44]. CC measures the linear correlation between the source image and the result. Higher CC indicates that the correlation is stronger [43].

From examining the results obtained from this experiment, the evaluation indexes of the fusion results obtained by using a deep learning curve are the best in SSIM, SF, MI, and STD. The result of CC is second only to NSCT. Overall, the fusion method based on deep learning is superior to the traditional fusion method.

### 3.2. Image Classification

**Figure 13:**
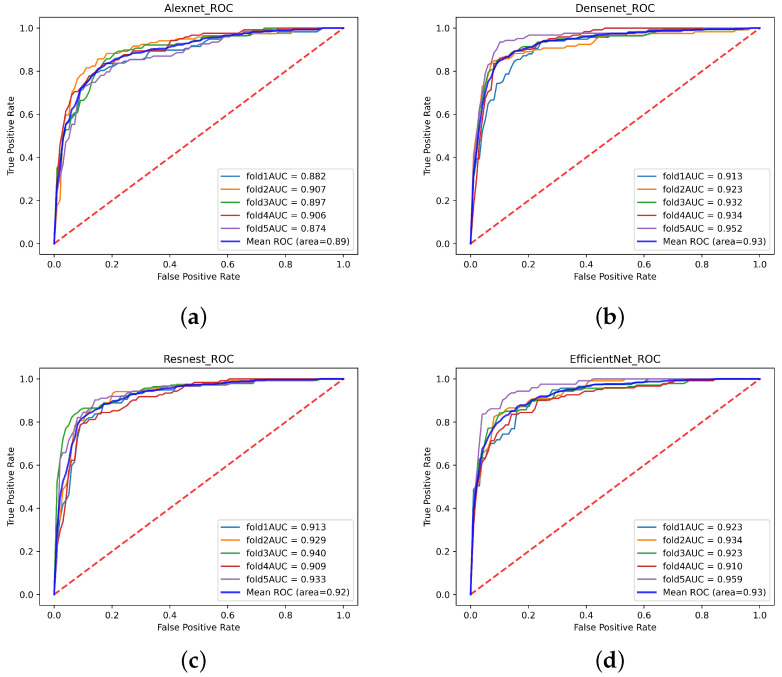
The receiver operating characteristic curve (ROC) curve of the single-modal data set. (**a**) Alexnet (**b**) Densenet (**c**) ResNeSt (**d**) Efficientnet.

**Figure 14:**
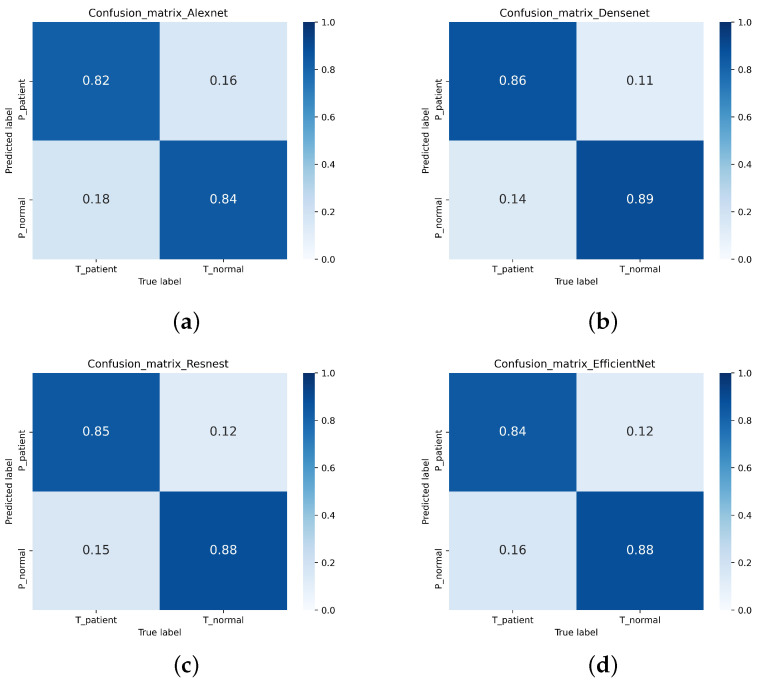
The confusion matrix of the single-mode MRI data set. (**a**) Alexnet (**b**) Densenet (**c**) ResNeSt (**d**) Efficientnet.

In this study, a single-modal MRI dataset containing 751 normal MRI and 621 Parkinson’s patients’ MRI from the PPMI database was used. After 5-fold cross-validation, the test accuracy of the single-modal MRI dataset on Densenet, ResNeSt, and Efficientnet were 87.76%, 86.37%, and 86.44%, respectively. However, only 83.31% of the single-modal MRI data set tested accuracy on Alexnet. Table 4 shows the Accuracy, Recall, Precision, Specificity, and F1-score of the single-modal MRI data set. The ROC of the single-modal MRI data set is shown in Figure 13. The confusion matrix of the single-modal MRI data set is shown in Figure 14.

**Figure 15:**
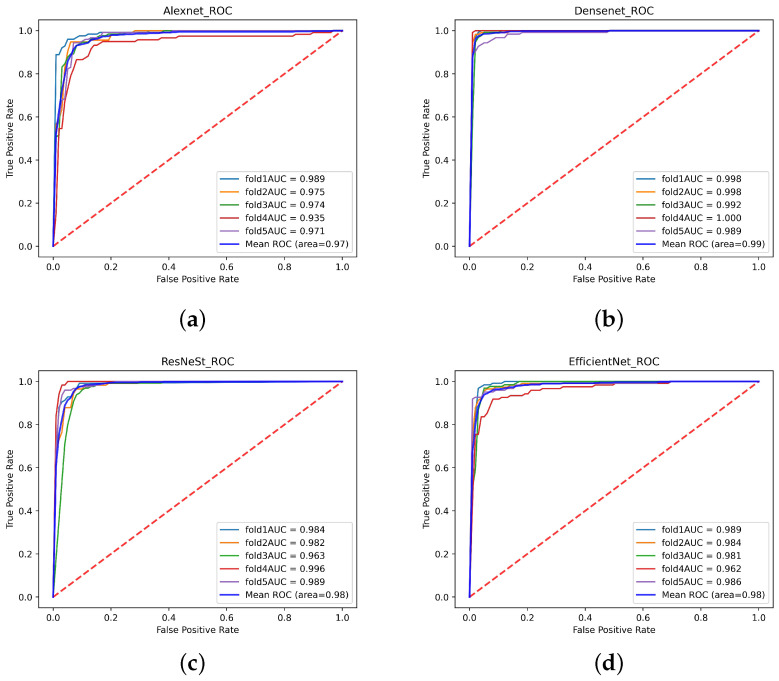
The ROC curve of the multi-modal data set. (**a**) Alexnet (**b**) Densenet (**c**) ResNeSt (**d**) Efficientnet.

**Figure 16:**
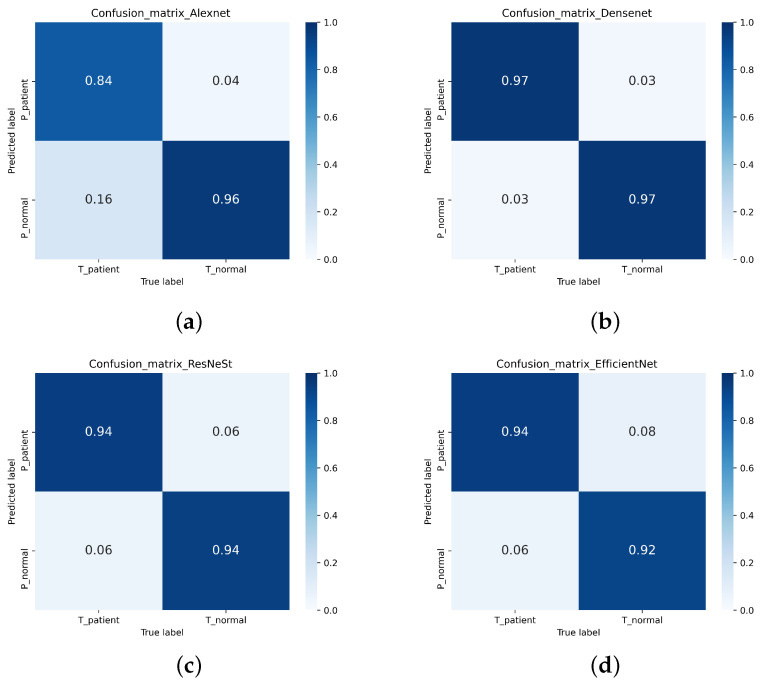
The confusion matrix of the multi-modal data set. (**a**) Alexnet (**b**) Densenet (**c**) ResNeSt (**d**) Efficientnet.

This experiment uses a multi-focus image fusion method based on a deep convolution neural network to retrieve a multi-modal dataset. The multi-modal dataset contains 736 healthy human images and 614 Parkinson’s patients. The test accuracies of the multi-modal data set on Densenet, ResNeSt, and Efficientnet were 97.19%, 94.15%, and 93.39% respectively. However, the test accuracy on Alexnet was 90.52%. Table 3 displays the Accuracy, Recall, Precision, Specificity, and $F_{1}$-Score indicators of the multi-modal data set training test results. The ROC of the multi-modal data set is shown in Figure 15. The confusion matrix of the multi-modal data set is shown in Figure 16.

In the past, Alexnet has been widely used in image classification when diagnosing PD. Sivaranjini [4] used the Alexnet network to classify the MRI of PD from the PPMI database. The accuracy of the experiment was achieved at 88.9%. In this investigation, Densenet, ResNeSt, and Efficientnet have not been applied to classify MRI of PD. In this article, the accuracy is discussed using Alexnet as a comparison. In this experiment, Alexnet, Densenet, ResNeSt, and Efficientnet were used to classify the PD single-modal MRI dataset and multi-modal fusion dataset.

In this experiment, Densenet, ResNeSt, and Efficientnet were used to classify the single-modal MRI dataset. The test accuracy from these results is greater than Alexnet. In terms of Recall, Precision, Specificity, and $F_{1}$-Score on these three networks, they are also better than Alexnet. This proves that these three networks have much more impact on the classification of Parkison’s images than the Alexnet network.

Densenet’s advantage is the utilization of small network parameters, which allows the feature transfer to be more effective, which improves the efficiency of training. The ResNeSt is divided into multiple feature groups. Each group is then divided into multiple sub-blocks. The sub-blocks greatly improve the efficiency of the network. The Efficientnet model proposes a compound scaling method, allowing the efficiency of training to be significantly improved. With these factors considered, the test accuracy and image classification of Densenet, ResNeSt, and Efficientnet are superior to Alexnet.

From the experiment results, Alexnet classified the single-modal MRI data set with an accuracy of 83.31%. The results from Alexnet are less than ideal from [4]. However, the multi-modal data set is 90.52%, which is better than the 88.9% in [4] and the result of single-modal MRI. In addition, the accuracy rates from Densenet, ResNeSt, and Efficientnet on the multi-modal dataset are also better than the single-modal MRI dataset’s counterpart. Therefore, image fusion can improve the accuracy of Parkinson’s image classification.

This paper used a multi-focus image fusion method based on a deep convolutional neural network to fuse images. Before this, focus detection, initial segmentation, and consistency verification processes are used. This method can retain the characteristics of the original image. Compared with the single-modal MRI dataset, the multi-modal dataset contains the characteristics of the PET image. Therefore, the accuracy obtained on the multi-modal dataset is higher.

## 4. Conclusions

The experimental results showed that using image fusion and convolution neural networks to classify images had high accuracy in classifying Parkinson’s images. When the results are compared with Alexnet, the three networks used in this paper have better results in the diagnosis of PD. Therefore, Densenent, ResNeSt, and Efficientnet are the ideal methods for classifying PD images. The test accuracy of multi-modal images fused by the multi-focus image fusion method based on the deep convolution neural network is significantly better than the single-mode MRI images on the neural network. This result shows that the fusion method has great significance in the diagnosis of PD, resulting in improving the accuracy of Parkinson’s image classification.

## Figures and Tables

**Table 1:** Data classification.

	True Patient	True Normal
Predict Patient	TP	FP
Predict Normal	FN	TN

**Table 2:** Confusion matrix.

	True Patient	True Normal
Predict Patient	$\frac{TP}{TP+FN}$	$\frac{FP}{FP+TN}$
Predict Normal	$\frac{FN}{TP+FN}$	$\frac{TN}{FP+TN}$

**Table 3:** The results of image fusion.

	SSIM	SF	MI	STD	CC
LP	0.8176	4.14	5.466	38.81	0.5825
RP	0.7883	4.15	5.431	37.90	0.6039
CVT	0.7860	3.96	5.458	35.64	0.6411
NSCT	0.7939	4.04	5.468	35.94	0.6360
This paper	0.8189	4.26	6.338	63.27	0.6350

**Table 4:** Experimental results.

CNN	Dataset	Accuracy	Recall	Precision	Specificity	$F_{1}$-Score
Alexnet	Single-modal	83.31%	81.87%	81.36%	84.95%	81.58%
	Multi-modal	90.52%	83.74%	94.79%	87.65%	88.90%
Efficientnet	Single-modal	86.44%	84.36%	85.46%	87.19%	84.88%
	Multi-modal	93.39%	94.43%	91.36%	95.45%	92.79%
ResNest	Single-modal	86.37%	84.60%	85.26%	87.29%	84.83%
	Multi-modal	94.15%	94.36%	93.07%	95.25%	93.63%
Densenet	Single-modal	87.76%	86.45%	86.49%	88.86%	86.78%
	Multi-modal	97.19%	97.09%	96.79%	97.59%	96.91%

## Data Availability

Data used in the preparation of this article were obtained from the Parkinson’s Progression Markers Initiative (PPMI) database in 20 March 2021 (https://www.ppmi-info.org/access-data-specimens/download-data). For up-to-date information on the study, visit www.ppmi-info.org.
